# T cell activation and cardiovascular risk in type 2 diabetes mellitus: a protocol for a systematic review and meta-analysis

**DOI:** 10.1186/s13643-018-0835-1

**Published:** 2018-10-20

**Authors:** Tawanda M. Nyambuya, Phiwayinkosi V. Dludla, Bongani B. Nkambule

**Affiliations:** 1 School of Laboratory Medicine and Medical Sciences (SLMMS), College of Health Sciences, University of KwaZulu-Natal, Durban, South Africa; 2 Department of Life and Environmental Sciences, Polytechnic University of Marche, Ancona, Italy; 3 Biomedical Research and Innovation Platform, Medical Research Council, Tygerberg, South Africa

**Keywords:** Cardiovascular diseases, Inflammation, T cell activation and exhaustion, Type 2 diabetes mellitus

## Abstract

**Introduction:**

The burden of non-communicable diseases such as type 2 diabetes mellitus (T2DM) and cardiovascular diseases (CVDs) has drastically increased in developing countries over the years. Although recent evidence points to chronic immune activation to be a significant aspect in the pathogenesis and development of T2DM and CVDs, the exact role of T cells is not fully understood. Therefore, we aim to investigate T cell function and cardio vascular risk in T2DM. In addition, the therapeutic effect of blood glucose-lowering drugs to reverse hyperglycaemia induced T cell dysfunction and myocardial infarction will be reviewed.

**Methods:**

This will be a systematic review and meta-analysis of published studies assessing T cell activation and cardiovascular risk in adults with T2DM. The search strategy will include medical subject headings (MeSH) words for PubMed/MEDLINE database. The search terms will also be adapted to grey literature, Embase and Cochrane Central Register of Controlled Trials electronic databases. Studies will be independently screened by two reviewers using predefined criteria. Relevant eligible full texts will be screened and data will be extracted. Data extraction will be performed using a pre-piloted structured form. To assess the quality and strengths of evidence across selected studies, the Grading of Recommendations Assessment Development and Evaluation approach will be used. The Cochran’s Q statistic and the *I*^2^ statistics will be used to analyse statistical heterogeneity between studies. If included studies show substantial level of statistical heterogeneity, a random-effects meta-analysis will be performed using R statistical software.

**Discussions:**

This review will not require ethical approval, and the findings will be disseminated through peer-reviewed publication and conferences. Although other previous studies have reported deregulated T cell function in hyperglycaemia, the underlying mechanisms remain controversial. However, evidence suggests that T cells may be a key component in the development of T2DM and CVDs as its complication. Furthermore, they are a potential diagnostic and therapeutic target in the management of the disease.

**Systematic review registration:**

PROSPERO CRD42018099745

**Electronic supplementary material:**

The online version of this article (10.1186/s13643-018-0835-1) contains supplementary material, which is available to authorized users.

## Background

In an era of rapid urbanisation and modernisation, the burden of non-communicable diseases has drastically increased worldwide, especially in developing countries [1, 2]. Of particular interest is type 2 diabetes mellitus (T2DM), a low-grade chronic inflammatory condition that is characterised by hyperglycaemia (high blood glucose level), insulin resistance and chronic activation of T cells [3–5]. Individuals living with T2DM have elevated levels of pro-inflammatory cytokines that may lead to immune dysfunction and increased risk of cardiovascular diseases (CVDs) [6–9]. Notably, the risk of morbidity and mortality due to CVDs is over fourfold higher in individuals with T2DM, compared to normoglycaemics [10].

The bidirectional relationship between T2DM and inflammation has been well described and involve the role of inflammation in causing both insulin resistance (IR) and hyperglycemia, which in turn further exacerbate inflammation [11–14]. For instance, chronic hyperglycaemia triggers activation of several metabolic and inflammatory pathways which include the aldose reduction pathway [15], advanced glycated end products (AGE) pathway [16], reactive oxygen intermediate pathway [17] and protein kinase C (PKC) pathway [11]. Furthermore, the AGE pathway modulates the nuclear factor-kappa B (NF-kB), phosphatidylinositol 3 kinase/protein kinase B (PI3K/Akt) and mitogen-activated protein kinase (MAPK) pathways resulting in further amplification of pro-inflammatory signals [11, 12]. This chronic exposure to proinflammatory mediators leads to the activation of cytokine signalling proteins which competes with insulin for binding sites and ultimately blocks the insulin signalling receptor resulting in the development of IR and hyperglycaemia [18].

Obesity-induced inflammation and insulin resistance play an important role in the pathogenesis of T2DM. The increased release of interleukin-6 (IL-6) and tumour necrosis factor alpha (TNFα) in individuals with T2DM enhances IR by inhibiting the activity of lipoprotein lipase which is responsible for the hydrolysis of triglycerides into free fatty acids [19, 20]. This reduces the uptake of glucose uptake by adipocytes. Furthermore, in obese T2DM individuals, the adipose tissue becomes hypertrophic and this triggers the production of proinflammatory cytokines and chemokines which attract immune cells [13]. This process causes the infiltration of innate immune cells such as pro-inflammatory macrophages (M1) into adipose tissue, moreover the switching of resident anti-inflammatory macrophages (M2) to M1 subtype [13]. These changes then lead to the initiation of an adaptive immune response. Infiltration of CD4^+^ T cells into the adipose tissue and their subsequent activation by adipocytes expressing major histocompatibility complex (MHC) class II has been implicated in the early stages of IR in obesity [21]. In addition, during the development of obesity, there is infiltration of B cells and their subsequent production of pathogenic antibodies in adipose tissue which leads to the activation of M1 macrophages and T cells and ultimately the development of IR [22].

It is well documented that chronic hyperglycaemia dysregulates T cell function [23, 24]; however, the underlying mechanisms remain controversial. In fact, contradictory findings of both elevated [13, 25–27] and decreased [28] levels of T cell activation have been reported in hyperglycaemic individuals. Furthermore, previous studies highlight the role of hyperglycaemia in activating pro-inflammatory T helper (Th) subsets [14, 25, 29]. Decreased expression of interleukin 2 receptor (CD25) on activated T cells has been reported in individuals with T2DM [30]. This may be indicative of a loss of the natural regulatory mechanism mediated by T cells in T2DM which further exacerbates T cell activation and inflammation. In contrast, others suggest that hyperglycaemia inhibits T cell activation by disrupting calcium transduction signalling [28]. Therefore, evidence on T cell function in metabolic diseases remains inconclusive.

The involvement and role of T cells in myocardial function and dysfunction has been well described. For instance, lymphocyte-deficient (RAG1 KO) mice revealed significantly smaller infarct sizes compared to the wild-type mice [31]. However, reconstitution of RAG1 KO mice by adoptive transfer of CD4^+^ T cells reversed this protection and showed an increase in the infarct sizes, therefore suggesting that CD4^+^ T cells promote myocardial ischaemia-reperfusion injury. A study on patients with acute coronary syndromes (ACS) reported a significant reduction in the number of regulatory T cells (Tregs) compared to the group of individuals with normal coronary arteries [32]. Furthermore, the study reported compromised function activity of Tregs in ACS compared to the control group. These findings implicate T cell activation and inability to suppress T cell function in the development of ACS.

Current T2DM drugs have been proven to be highly effective in the management of hyperglycaemia albeit offering limited cardio-protection [33, 34]. One of these drugs is metformin, a first-line oral anti-diabetic drug which lowers blood glucose levels through direct suppression of hepatic glucose production and the activation of adenosine-monophosphate-activated protein kinase (AMPK). Interestingly, AMPK regulates cellular energy homeostasis and T cell differentiation [35, 36]. However, the exact impact of metformin on T cell function and cytokine release is not fully understood. A study by Zarrouk et al. reported on a decreased expression of CD25 and activation inducer molecule (CD69) in antigen-activated T cells exposed to metformin when compared to the control group [35]. Furthermore, the study reported failure of metformin-treated T cells to express transferrin receptors and inability to increase glucose uptake [35], thus suggesting alterations in T cell function during metformin treatment, subsequent to the aggravation of a pro-inflammatory response.

This systematic review will for the first time assess available literature on the effect of hyperglycaemia on T cell function, including activation and exhaustion. Furthermore, it will assess the role of T cells in inducing myocardial dysfunction and the therapeutic intervention of glucose-lowering drugs to reverse these effects.

### Research question

What is the role of T cell activation in the development of cardiovascular diseases in T2DM? Furthermore, what is the effect of anti-hyperglycaemic drugs on T cell function?

### Objectives

1. To investigate T cell function in T2DM
2. To evaluate Th_1_ and Th_2_ T cell function in treated individuals with T2DM and their association with increased risk of CVDs.
3. To assess whether metformin is effective in reversing hyperglycaemia-induced T cell activation and protect against myocardial injury.

## Methods

### Protocol and registration

The systematic review protocol has been prepared according to the Preferred Reporting Items for Systematic Review and Meta-Analysis Protocols (PRISMA-P) 2015 guidelines [37].

The protocol has been submitted on PROSPERO for registration. A detailed checklist for this review protocol is provided as PRISMA-P checklist (see Additional file 1 attachment).

### Eligibility criteria

This study will include both observational and interventional studies inclusive of cross-sectional and case-control studies with a clearly defined control population. In addition, randomised controlled trials (RCTs) and retrospective and prospective cohort studies with defined time points highlighting data points before and after intervention will be included. Animal studies, case studies and case reports will be excluded from the review. Furthermore, we will also include studies that report the exclusion of participants using steatogenic medications or drugs that interfere with the immune system. Studies that include pregnant women and patients with a known history of T cell malignancy will be excluded.

### Participants

Studies on T cell function in adults (> 18 years) with both T2DM and CVDs will be included.

### Interventions

We will consider studies that have clearly defined the anti-hyperglycaemic drugs used.

### Comparators

The primary comparisons will include:

1. Individuals with T2DM vs the normoglycaemic group (control)
2. Individuals with T2DM on treatment vs control group
3. Individuals with T2DM on treatment vs individuals with T2DM not on treatment group

### Outcomes

Primary outcomes will include:

1. T cell activation reported as mean percentage expression or mean florescence intensity of HLA-DR, CD38, CD69 and CD95 or Th_1/2_ cytokine secretion.
2. T cell exhaustion reported as mean percentage expression or mean florescence intensity of PD-1.
3. Cardiovascular events associated with T2DM.
  - Coronary artery events: fatal myocardial infarction, non-fatal myocardial infarction, unstable angina and stable angina.
  - Cerebrovascular events: fatal stroke, non-fatal stroke (ischaemic or haemorrhagic), transient ischaemic attack and vascular events.

Secondary outcomes will include:

1. For T2DM: insulin resistance, impaired glucose tolerance and increased glycated haemoglobin (HbA1c).
2. For cardiovascular risk (total cholesterol, high-density lipoprotein cholesterol level, systolic blood pressure, dyslipidaemia and smoking).
3. For T cell activation: increased biomarker levels of inflammation (CRP), leucocytosis and high erythrocyte sedimentation rate (ESR).

### Search strategy

The systematic search will be conducted without any language restrictions. However, for none English articles, only those google translatable will be searched. The search strategy will consist of the following major keywords and their respective synonyms: type 2 diabetes mellitus, hyperglycaemia, inflammation, CVDs, T cell activation and exhaustion. For each keyword, multiple synonyms will be searched in the title or abstract. In addition, the reference lists of selected studies will be scanned to identify relevant literature. A search strategy will be developed using medical subject headings (MeSH) words and their respective synonyms on MEDLINE (see Additional file 2). The search strategy will also be adapted to grey literature, Embase and Cochrane Central Register of Controlled Trials electronic databases and will be peer-reviewed by a librarian specialist.

### Study selection

A standard data extraction sheet will be used to extract data from the screened and selected studies. The appraisal worksheet will enable the extraction of the following information: aims and objectives of the study, study population, country where the study was conducted, funding source, participant demographics, year published, study type, treatment drugs used, methods and techniques used to assess T cell activation and statistical methods used and limitations of the study. The study selection process will be carried out independently by two reviewers (TMN and BBN) to eliminate any discrepancies and inconsistencies regarding reviewers’ inclusion and exclusion of studies. In case of disagreements, PVD will be consulted for arbitration. The appraisal of studies will be documented using Microsoft Excel, and the V.1.18 Mendeley reference manager (Elsevier, Amsterdam, Netherlands) will be used to identify duplicates.

### Data collection process

To ensure effective data collection from the selected studies, a pre-piloted structured form will be used to collect data items (listed below). The titles, abstracts and full texts yielded by the search against the inclusion criteria will be used to collect relevant data. To minimise data entry errors, selected studies will be carefully and independently assessed by two different authors (TMN and PVD) to extract relevant data. The other author (BBN) will be consulted for arbitration in case of any disagreements.

### Data items

The data items that will be extracted include the name of the authors, year of publication, cohort sample size and duration of follow-up. In addition, participant characteristics such as average age, gender ratio, glucose metabolic profile (blood glucose levels, glycated haemoglobin and fasting insulin) levels of inflammatory biomarkers (C-reactive protein and cytokines), levels of T cell activation and exhaustion markers and their treatment status will also be extracted. In addition, details related to the assays used to measure the levels of T cell function (activation and/or exhaustion) as well as the techniques used will be extracted. Since CVD is a broad category, we will extract the type of cardiovascular event reported from each respective study. Furthermore, the CVDs will be categorised into micro- and macrovascular diseases. The surrogate outcomes for T2DM will include insulin resistance and impaired glucose tolerance that may be reported based on varying outcome measures. In cases where there are no reported data amputation techniques on priority outcomes and when the effect size cannot be calculated, the authors will be contacted for additional information.

### Data simplification

As a data simplification measure, studies that mention that participants were on diabetic treatment will be grouped as the treatment group irrespective of the drugs used and those with diabetes and not on treatment as the non-treatment group. Furthermore, the levels of T cell activation and exhaustion will be reported as a continuous variable and will be compared by calculating the standardised mean difference (SMD).

### Risk of bias in individual studies

The Cochrane risk of bias tool will be used to assess risk of bias in included randomised controlled trials [38]. The Joanna Briggs Institute (JBI) Critical Appraisal tools with specific checklists for non-randomised experimental studies will be used for other types of studies [39]. A judgement on the possible risk of bias of extracted information will be made based on each of the six domains. The judgement will be made independently by two reviewers (PVD and BBN) based on the criteria defined for judging the risk of bias. In instances where these two reviewers disagree, TMN will be consulted for arbitration. Furthermore, funnel plot analysis will be used to assess publication bias and the Harbord and Peters test will be used to test the funnel plot asymmetry.

### Data synthesis

The Cochran’s Q statistic [40] and the *I*^2^ statistics will be used to analyse statistical heterogeneity between studies [38]. An *I*^2^ value of > 25 will be considered as moderate or substantial heterogeneity [41]. If an efficient number of studies included are homogeneous in terms of extracted information, we will conduct a meta-analysis using R statistical Software (The R foundation for statistical computing, Vienna, Austria). The random-effects model will be used should there be significant levels of unexplained statistical heterogeneity [42]. In order to explore the sources of heterogeneity within the included studies, a subgroup analysis and meta-regression compare the study estimates from different study-level characteristics, which will include, age, gender, treatment drugs, reported measure of T cell activation (CD38, CD69, HLA-DR,CD95) and exhaustion (PD-1). Furthermore, data from clinical trials and observational studies will be analysed and used separately.

### Cumulative evidence

To assess the quality and strengths of evidence across selected studies, two independent reviewers (PVD and BBN) will review the studies using the Grading of Recommendations Assessment Development and Evaluation (GRADE) approach [43]. The approach will be implemented by the downgrading of studies based on several factors such as study limitations, indirectness of results and publication or reporting bias. The scores will only be upgraded in exceptional cases where individual judgments (inconsistency, indirectness, imprecision and publication bias) are of low risk. Ratings for each outcome will be categorised as high, moderate or low. This will then be followed by the rating of the overall quality. The findings will be summarised and presented in the summary of findings table.

## Discussion

Although other previous studies have reported immune dysfunction in a diabetic state, the involvement of adaptive immune response, particularly T cells, still remains limited and controversial. However, evidence suggests that T cells may be a key component in the development of T2DM and CVDs as its complication. Furthermore, they are a potential diagnostic and therapeutic target in the management of the disease. Therefore, the findings of this review will indicate novel avenues to explore at a molecular level in finding solutions in the management and treatment of diabetics. This in turn will help reduce the burden of diabetes and its complications on national health budgets.

## Additional files

Additional file 1: PRISMA-P-checklist. (DOC 81 kb)

Additional file 2: Search strategy. (DOCX 19 kb)

## Abbreviations

AGE: Advanced glycated end product
CD: Cluster of differentiation
CVDs: Cardiovascular diseases
DM: Diabetes mellitus
IR: Insulin resistance
T2DM: Type 2 diabetes mellitus
Th: T helper cells
