# Supplementary material for: T cell activation and cardiovascular risk in type 2 diabetes mellitus: a protocol for a systematic review and meta-analysis
Source: Syst Rev. 2018 Oct 20;7:167. doi: 10.1186/s13643-018-0835-1 (PMC6195734; doi:10.1186/s13643-018-0835-1)
Supplement: Supplementary file 2 — Search strategy. (DOCX 19 kb) [file 13643_2018_835_MOESM2_ESM.docx]

| **Concept 1:**  **Diabetes mellitus** | **Synonyms to be searched (MeSH or Text words)** |
| --- | --- |
| **PubMed** (hits=114 230)  “Diabetes mellitus, Type 2”[Mesh] | Diabetes mellitus |
|  | Glucose metabolism disorders |
|  | Hyperglycaemia |
|  | Metabolic diseases |
|  | Metabolic syndromes |

| **Concept 2:**  **T-lymphocytes** | **Synonyms to be searched (MeSH or Text words)** |
| --- | --- |
| **PubMed** (hits=114 230)  “T-lymphocytes”[Mesh] | T-cells |
|  | Th1/Th2 cells |
|  | CD4^+^ |
|  | CD8^+^ |
|  |  |

| **Concept 3:**  **Cardiovascular Diseases** | **Synonyms to be searched (MeSH or Text words)** |
| --- | --- |
| **PubMed** (hits=2 191 229)  “Cardiovascular diseases”[Mesh] | Heart diseases |
|  | Vascular diseases |
|  |  |
|  |  |
|  |  |

**Additional file 2: Search Strategy ran 06 June 2018**

**Combined Concept 1, 2 and 3 (PubMed hits = 51)**
